# A case of spontaneous mesenteric hematoma with diagnostic difficulty

**DOI:** 10.1186/s40792-020-00867-0

**Published:** 2020-06-01

**Authors:** Nobuhisa Tanioka, Hiromichi Maeda, Sachi Tsuda, Jun Iwabu, Tsutomu Namikawa, Mitsuko Iguchi, Kazuhiro Hanazaki

**Affiliations:** 1 Department of Surgery, Kochi Medical School Hospital, Kochi University, Kohasu, Oko-cho, Nankoku, Kochi 783-8505 Japan; 2 Department of Pathology, Kochi Medical School, Kohasu, Oko-cho, Nankoku, Kochi 783-8505 Japan

**Keywords:** Intraperitoneal hemorrhage, Mesenteric hematoma, Gastrointestinal stromal tumor, Calcification

## Abstract

**Background:**

Spontaneous mesenteric hematoma (SMH) is a rare condition characterized by intraperitoneal hemorrhage of unknown etiology. SMH without worsening of general status allows conservative management; however, patients showing chronological changes on imaging require surgical intervention to rule out possible malignancy.

**Case presentation:**

A 69-year-old man was referred to our hospital to evaluate an abdominal mass with no associated clinical symptoms. He had a history of chronic hepatitis C and diabetes mellitus. Six months earlier, computed tomography (CT) revealed a 75-mm tumor arising from the jejunum, suspected to be a gastrointestinal stromal tumor (GIST) of the small intestine. Following a further 6 months of observation, the patient was referred to our hospital. Abdominal contrast-enhanced CT revealed a well-defined heterogeneous round tumor with a maximum diameter of 75 mm adjacent to the upper jejunum. The tumor was accompanied by calcification at the periphery, with no evidence of augmentation over the prior 6 months. Diffuse lymphadenopathy was observed around the aorta and splenic artery. Under the diagnosis of GIST arising from small intestine, the patient underwent elective surgery. The resection revealed an elastic soft tumor at the mesentery adjacent to the upper jejunum with severe adhesion between the tumor and jejunum. The tumor origin was unclear; thus, we performed mesenteric excision and partial enterectomy without lymph node dissection.

The tumor was surrounded by fibrous capsular tissue containing massive hemosiderin deposits and cholesterol crystals showing partial calcification, resulting in a diagnosis of spontaneous hematoma of the mesentery.

**Conclusions:**

We report a case of SMH mimicking small intestinal GIST. It is difficult to diagnose long-established SMH because its radiological features change with time, and more case reports are needed to improve the accuracy of diagnosis.

## Background

Mesenteric hematoma is a rare condition resulting from localized bleeding in peripheral mesenteric vessels that is generally caused by abdominal trauma, postoperative complications, or aneurysm [1–3]. Previous studies identified several potential risks for mesenteric hematoma including pancreatitis, vasculitis, connective tissue disease, and anticoagulant mismanagement [4–6]. Spontaneous mesenteric hematoma (SMH) is generally defined as a mesenteric hematoma in the absence of these causative clinical and pathological findings.

A SMH patient present with nonspecific symptoms and diagnosis is generally made based on abdominal contrast-enhanced computed tomography (CT), ultrasound, or magnetic resonance imaging (MRI). SMH can be safely observed if the patient shows no sign of worsening general status or exacerbation of symptoms; however, the onset of diagnostic difficulty due to changes in the imaging findings over time requires surgical resection for both diagnosis and treatment.

## Case presentation

**Fig. 1:**
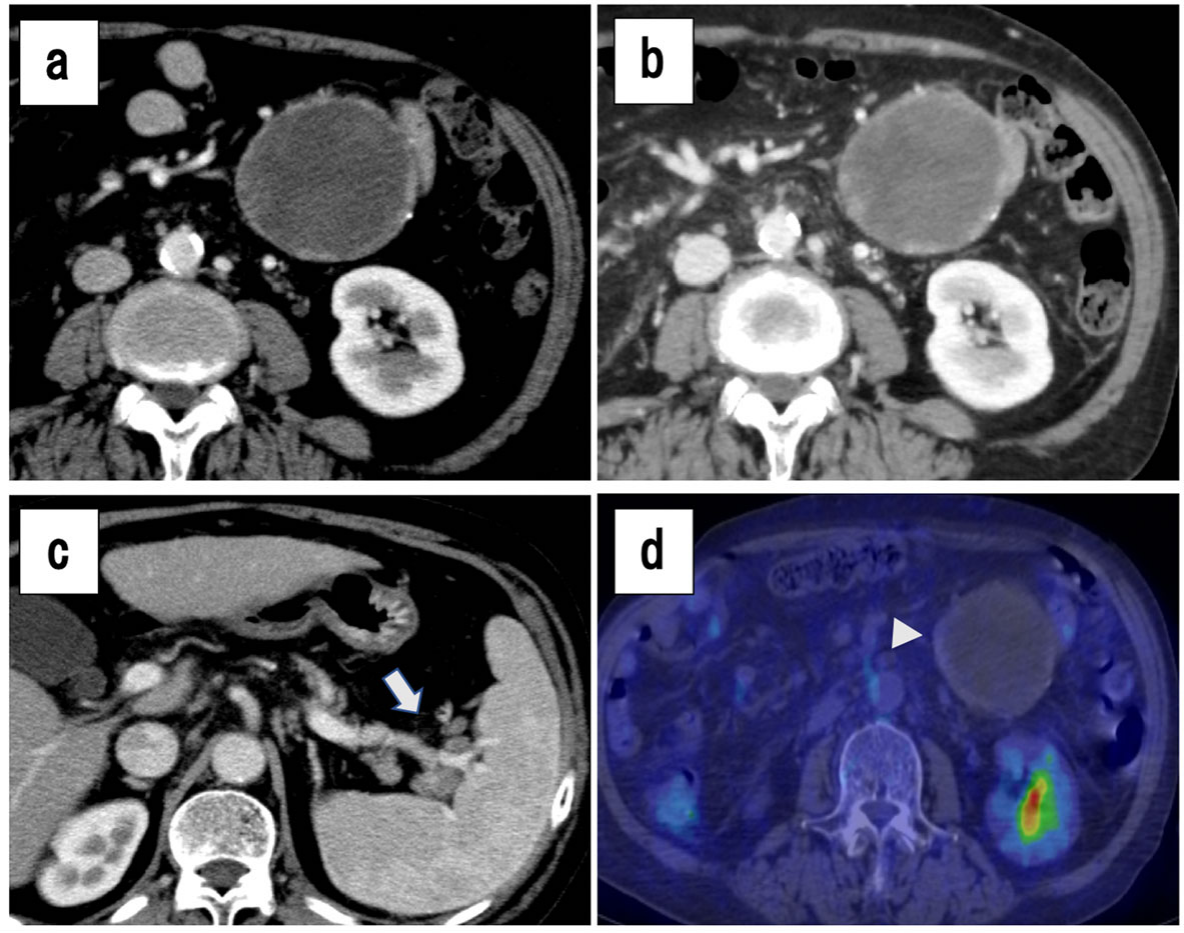
Abdominal contrast-enhanced computed tomography (CT) findings (**a–c**). **a** A well-defined, mixed-density, round tumor with a maximum diameter of 77 mm, adjacent to the upper jejunum. **b** The tumor was accompanied by calcification at part of the border. **c** Diffuse lymphadenopathy was detected at the paraaortic, splenic artery region, and splenic hilum area (arrow). **d** (18)F-FDG PET-CT showed slight accumulation (SUVmax 1.5–2.0) along the tumor border, corresponding to the calcified portion (arrowhead)

A 69-year-old man, without a remarkable family history, was referred to our hospital with an undiagnosed, asymptomatic abdominal mass. He had a history of chronic hepatitis C and diabetes mellitus, and generally used a proton pomp inhibiter and branched-chain amino acid formula without any anticoagulant. The patient was also a habitual drunkard. Six months earlier, his previous doctor diagnosed chronic hepatitis and detected a 75-mm tumor arising from the jejunum by CT imaging (Fig. 1a). The doctor suspected the tumor to be a GIST of the small intestine. The patient was referred to our hospital 6 months following the imaging results, but he presented no characteristic clinical symptoms such as abdominal pain, nausea, and melena. Physical examination revealed a hard-palpable mass in the left upper quadrant of the abdomen, and laboratory blood testing revealed the following findings: hemoglobin, 11.0 g/dL (normal range 13.4–17.4 g/dL); white blood cells, 1500/L (3600–9000/L); platelet count, 132 × 100/L (138–309 × 100/L); albumin, 3.0 g/dL (3.8–5.3 g/dL); total bilirubin, 0.9 mg/dL (0.2–1.2 mg/dL); aspartate transaminase (AST), 76 IU/L (10–35 IU/L); alanine transaminase (ALT), 61 IU/L (5–46 IU/L); alkaline phosphatase (ALP), 809 IU/L (115–359 IU/L); γ-glutamyl transpeptidase (GTP), 27 IU/L (16–73 IU/L); hepatitis C virus (HCV) 3 antibody, 46.5 U (< 1.0 U); and HCV-RNA 6.0 log IU/mL (< 1.2 log IU/mL). Other laboratory tests were within normal range, including a blood smear and tumor marker analysis (carcinoembryonic antigen, carbohydrate 19-9 and carbohydrate 125, soluble interleukin-2 receptor).

Abdominal contrast-enhanced CT revealed a well-defined, heterogeneous, round tumor, with a maximum diameter of 75 mm adjacent to the upper jejunum, with no worsening for 6 months (Fig. 1b, c). The tumor showed calcification at the periphery and there was diffuse lymphadenopathy around the aorta and splenic artery. Imaging by (18)F-fluorodeoxyglucose (FDG) positron emission tomography (PET)-CT showed slight accumulation (SUV_max_ 1.5–2.0) of FDG around the tumor, but no sign of FDG accumulation inside the tumor (Fig. 1d). The nuclear isotope also appeared in the diffuse lymphadenopathy, but the results were difficult to differentiate from nonspecific accumulation due to chronic inflammation.

Based on the findings to this point, we diagnosed the tumor as GIST arising from jejunum and performed a laparotomy for the purpose of diagnosis and treatment. An elastic soft tumor was located at the mesentery adjacent to the upper jejunum with severe adhesion between the tumor and jejunum. The tumor origin was unclear; thus, we performed mesenteric excision and partial enterectomy without lymph node dissection.

**Fig. 2:**
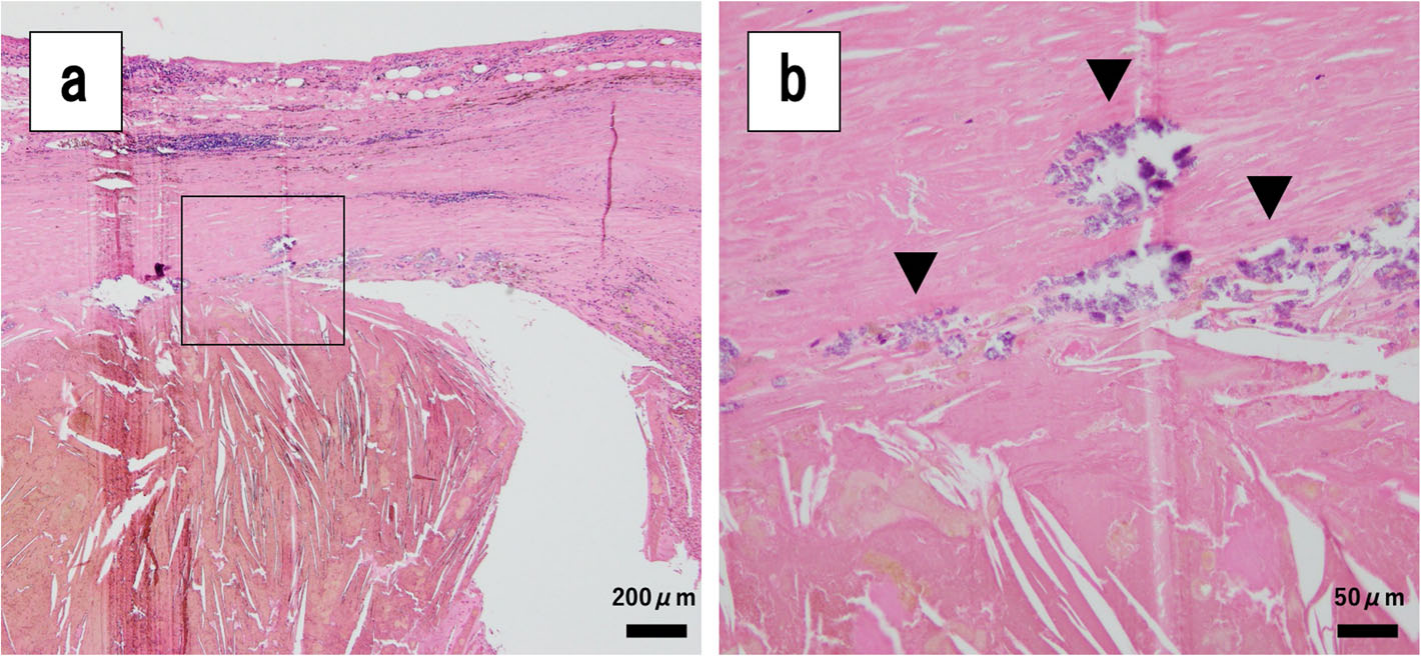
Pathological findings. **a**, **b** The tumor was surrounded by a fibrous capsule, containing massive hemosiderin deposits and cholesterol crystals showing partial calcification (arrowhead). The cystic wall contained no elastic fibers, vessels, or epithelial cells, indicating it was a pseudocyst. The tumor was located in mesenteric fat tissue and in the jejunal serosa, with no atypical cells at the jejunal mucosal surface

Histological analysis of the resected tumor showed a cystic lesion surrounded by fibrous capsular tissue containing extensive hemosiderin deposits and cholesterol crystals with partial calcification (Fig. 2a). The cystic wall contained no obvious elastic tissue, vessels, or epithelial cells, indicating that the lesion was a pseudocyst. Tumor was also located in the mesenteric fat tissue at the jejunal serosa, and there were no atypical cells in the jejunal mucosal surface (Fig. 2b). Immunohistochemistry showed negative findings for S-100, DOG-1, and 1α-SMA. The resected specimen also showed mesenteric hematoma, but histopathology did not reveal the source of bleeding such as aneurysm or vasculitis. There are several diseases that may cause mesenteric hematoma, such as Ehlers-Danlos syndrome, Osler-Weber-Rendu disease, polyarteritis nodosa, Behçet disease, and several bleeding diatheses. Although we did not thoroughly investigate the presence or absence of these conditions, we finally diagnosed it as SMH because there were no history or clinical signs suggesting of these conditions. The patient was discharged on POD 11 with no significant complications.

## Discussion

SMH is a rare condition involving intraperitoneal hemorrhage [7]. The most common clinical symptom is abdominal pain, the severity of which depends on the location and size of the tumor [8, 9]. Nausea or constipation may occur if the tumor is large enough to compress the digestive tract [10]. Nonspecific symptoms make it difficult to reach a diagnosis from medical interview, and SMH is usually identified by abdominal contrast-enhanced CT, ultrasound, or magnetic resonance imaging (MRI) [8].

To the best of our knowledge, only 10 reported cases of SHM accompanied by comprehensive and publicly available data appear by MEDLINE searching of English literature (Table 1) [8–17], and 5 of these were reported from Asia. Together, these cases reveal two important findings; it is relatively easy to reach the diagnosis of hematoma in the early phase from onset, and aspiration biopsy should not be performed for the diagnosis. Unlike our case, the other 10 cases involved different symptoms such as abdominal pain, nausea, and diarrhea, while 6 patients had a history of sudden onset within 1 week, and 5 were initially diagnosed as hematoma based on enhanced CT or evidence of anemia progression. In 7 patients with stable general status who were observed conservatively, 1 showed no shrinkage of tumor following 60 days of observation and underwent elective surgery [12]. Another patient underwent biopsy during the observation period because malignant disease could not be excluded by initial imaging, resulting in rebleeding and emergency surgery [15]. We thus consider that tumor puncture is not recommended in any situation due to the possibility of rebleeding and peritoneal dissemination.

**Table 1:** Previous reports of spontaneous mesenteric hematoma

No	Authors		Age (y)	Sex	Clinical symptoms	Duration from onset (day)	Tumor diameter (mm)	Anemia	Extravasation	Initial diagnosis	Embolization	Observation	Observation period (day)	Volume reduction	Surgery
1	Raghavendra et al (ref [11])	1982	37	F	Abdominal pain	7	70	NA	ー	Hematoma	None	Yes	330	Yes	None
2	Singla et al (ref [10])	1989	48	F	Abdominal pain, Constipation, Vomiting	3	90	Yes	ー	NA	None	Yes	NA	ー	Yes
3	Aoki et al (ref [12])	1990	47	M	Abdominal pain	14	40	None	None	Hematoma Malignancy	None	Yes	60	None	Yes
4	Parker et al (ref [8])	2012	54	M	Abdominal pain, Nausea, Diarrhea	1	180	None	None	Hematoma	None	Yes	90-120	Yes	None
5	Ono et al (ref [13])	2013	64	M	Abdominal pain, Nausea	Unknown	NA	None	None	NA	None	Yes	21	Yes	None
6	Ashrafian et al (ref [14])	2014	44	F	Abdominal pain, Constipation	Within 30	200	Yes	None	NA	None	No	0	ー	Yes
7	Sutton et al (ref [15])	2015	67	M	Abdominal pain	2	100	None		Hematoma Malignancy	None	Yes	30	ー	Yes
8	Shikata et al (ref [16])	2016	75	M	Melena	1	30	Yes	Yes	Hematoma	None	No	0	ー	Yes
9	Hirano et al (ref [9])	2018	71	M	Diarrhea, Melena	Unknown	100	Yes	Yes	Hematoma	Yes	No	0	ー	Yes
10	Bekki et al (ref [17])	2019	90	M	Abdominal pain	1	120	Yes	Yes	Hematoma	None	Yes	2	Yes	Yes
11	Tanioka et al	2019	69	M	None	Unknown	75	Yes	None	GIST	None	Yes	180	None	Yes

Takashimizu et al. [18] reviewed 20 cases of SMH published in Japanese and precisely analyzed the imaging findings. Their results suggested that the CT imaging of hematoma changes with time, and it is particularly difficult to diagnose SMH after a long time has passed from onset. On standard CT imaging, hematoma showed 70–90 Hounsfield Unit (HU) with high- and iso mixed-density lesions during 0–14 days from onset, 20–30 HU with high- and low mixed-density lesion during 14–28 days, and low-homogeneous lesion after 28 days. On enhanced CT imaging, hematoma showed well-defined lesion with enhanced periphery during 14–28 days, and sometimes accompanied by an enhanced septal wall like polycystic lesion after 28 days. Since malignant disease could not be excluded, 6 patients underwent surgery; these all belonged to the group of over 28 days since onset or unknown onset. This course was chosen because it is difficult to distinguish chronic-course hematoma from abscess or other cystic tumors, particularly when it forms a polycystic appearance. It is also important to distinguish SMH from GIST of small intestine or mesenteric lymphoma due to the most common site.

In our case, the mesenteric hematoma was accompanied by an enhanced periphery with calcification, but we did not find this feature in previous reports. It is not surprising that chronic hematoma becomes calcified, since similar findings are reported regularly with long-term hemorrhage such as chronic subdural hematoma [19, 20]; however, such a clinical picture did not rule out GIST in our patient at that time.

The usefulness of tumor calcification pattern for distinguishing SMH from small intestinal GIST remains unclear, largely because few reports have investigated the calcified hematoma with the exception of chronic subdural hematoma. Such calcification is estimated to occur in 0.3–2.7% of cases with chronic subdural hematoma, identified by high density at the lesion rim by CT [21]. On the other hand, calcified GIST is also rare (1.5–12%) and commonly forms as nodular or focal calcifications [22, 23], which sometimes form an extensive pattern [24]. To our knowledge, no related report focuses on the pattern of calcified GIST, although while tumor accompanied by calcification from the periphery raises the possibility of hematoma, it cannot completely rule out a GIST diagnosis.

## Conclusion

In summary, we described a case of SMH mimicking small intestinal GIST. It is difficult to diagnose long-term SMH because its radiological features change over time, and differ among cases. Further case reports are needed to improve the accuracy of diagnosing SMH.

## Data Availability

The dataset supporting the conclusions of this article is included within the article.

## Abbreviations

SMH: Spontaneous mesenteric hematoma
GIST: Gastrointestinal stromal tumor
CT: Computed tomography
AST: Aspartate transaminase
ALT: Alanine transaminase
ALP: Alkaline phosphatase
GTP: γ-glutamyl transpeptidase
HCV: Hepatitis C virus HCV
FDG: Fluorodeoxyglucose
PET: Positron emission tomography
DOG-1: Discovered on GIST
α-SMA: α-smooth muscle actin
MRI: Magnetic resonance imaging
SUV: Standardized uptake value
HU: Hounsfield Unit
